# The Lectin Receptor Kinase LecRK-I.9 Is a Novel *Phytophthora* Resistance Component and a Potential Host Target for a RXLR Effector

**DOI:** 10.1371/journal.ppat.1001327

**Published:** 2011-03-31

**Authors:** Klaas Bouwmeester, Mara de Sain, Rob Weide, Anne Gouget, Sofieke Klamer, Herve Canut, Francine Govers

**Affiliations:** 1 Laboratory of Phytopathology, Plant Sciences Group, Wageningen University, Wageningen, The Netherlands; 2 Centre for BioSystems Genomics (CBSG), Wageningen, The Netherlands; 3 UMR 5546 CNRS-Université Paul Sabatier-Toulouse III, Castanet-Tolosan, France; University of Melbourne, Australia

## Abstract

In plants, an active defense against biotrophic pathogens is dependent on a functional continuum between the cell wall (CW) and the plasma membrane (PM). It is thus anticipated that proteins maintaining this continuum also function in defense. The legume-like lectin receptor kinase LecRK-I.9 is a putative mediator of CW-PM adhesions in Arabidopsis and is known to bind *in vitro* to the *Phytophthora infestans* RXLR-dEER effector IPI-O via a RGD cell attachment motif present in IPI-O. Here we show that LecRK-I.9 is associated with the plasma membrane, and that two T-DNA insertions lines deficient in LecRK-I.9 (*lecrk-I.9*) have a ‘gain-of-susceptibility’ phenotype specifically towards the oomycete *Phytophthora brassicae*. Accordingly, overexpression of *LecRK-I.9* leads to enhanced resistance to *P. brassicae*. A similar ‘gain-of-susceptibility’ phenotype was observed in transgenic Arabidopsis lines expressing *ipiO* (35S-*ipiO1*). This phenocopy behavior was also observed with respect to other defense-related functions; *lecrk-I.9* and 35S-*ipiO1* were both disturbed in pathogen- and MAMP-triggered callose deposition. By site-directed mutagenesis, we demonstrated that the RGD cell attachment motif in IPI-O is not only essential for disrupting the CW-PM adhesions, but also for disease suppression. These results suggest that destabilizing the CW-PM continuum is one of the tactics used by *Phytophthora* to promote infection. As countermeasure the host may want to strengthen CW-PM adhesions and the novel *Phytophthora* resistance component LecRK-I.9 seems to function in this process.

## Author Summary

*Phytophthora* species are notorious plant pathogens which cause a variety of devastating crop diseases. *Phytophthora* pathogens secrete a plethora of effector proteins, several of which are known to interact with receptors in the host cell thereby either activating or suppressing defense responses. Unlike animals, plants lack an adaptive immune system; however, they are not defenseless and have acquired other mechanisms to withstand pathogens. Receptor proteins play important roles in sensing alterations at the plant cell wall and in mediating responses upon pathogen attack. This paper focuses on the Arabidopsis lectin receptor kinase LecRK-I.9, a mediator of cell wall – plasma membrane (CW-PM) adhesions that is known to bind *in vitro* to the *Phytophthora infestans* effector IPI-O via the cell attachment motif RGD. T-DNA mutants deficient in *LecRK-I.9* and transgenic Arabidopsis lines expressing *ipiO1* were found to behave as phenocopies. Both show a ‘gain-of-susceptibility’ phenotype towards the Arabidopsis pathogen *Phytophthora brassicae* and are disturbed in callose deposition. Overall, the results suggest that destabilizing the CW-PM continuum is a strategy for *Phytophthora* to promote infection. As countermeasure, the host may want to strengthen CW-PM adhesions, and the novel resistance component LecRK-I.9 apparently functions in this process.

## Introduction

Plants deploy multiple strategies to defend themselves against pathogen attack. A key step is the perception of pathogen molecules in order to activate various defense responses. During infection pathogens produce microbe-associated molecular patterns (MAMPs) or elicit the production of host-derived damage-associated molecular patterns (DAMPs). These signals are recognized by the plant via so-called pattern recognition receptors (PRRs) and trigger cell wall-associated defenses, such as the production of antimicrobial compounds and cell wall strengthening [1], [2]. An active defense response is, however, dependent on a functional continuum between the cell wall (CW) and the plasma membrane (PM) [3]. When this continuum is disturbed the effectiveness of defense is lost. Destabilizing the CW-PM continuum to circumvent recognition could therefore be a strategy for a pathogen to promote infection.

In animals, cell adhesion is mediated by integrins, membrane-spanning receptors whose ligands are extracellular matrix (ECM) proteins carrying the cell-attachment motif Arg-Gly-Asp (RGD) [4]. Other proteins or peptides comprising the motif RGD can act as integrin antagonists and as such interfere with integrin-related functions, including cell adhesion and proliferation [5], [6]. Examples are certain viral, bacterial and fungal proteins [7]–[9], and various snake venom disintegrins [10].

Also plant pathogens produce proteins carrying a RGD motif. Well-studied is PtrToxA, a secreted proteinaceous toxin of the foliar wheat pathogen *Pyrenophora trititci-repentis*, which induces cell death in toxin-sensitive wheat mesophyll cells [11]. Internalization of PtrToxA is dependent on its RGD-containing solvent loop, which is largely identical to the integrin-binding RGD loop of the mammalian ECM glycoprotein vitronectin [12], [13]. PtrToxA proteins with mutations in the RGD-loop were found to be less toxic due to impaired internalization. Toxicity of PtrToxA was also reduced when RGD peptides were added as competitor and this supports the hypothesis of a RGD-dependent receptor-mediated endocytosis [13].

Several studies have shown that exogenously applied RGD peptides can have a strong disrupting effect on plant cells. For example, in cell suspensions of soybean and Arabidopsis, and in onion epidermal cells RGD peptides caused loss of CW-PM adhesions in a concentration dependent manner, whereas RGE or DGR peptides did not show this effect [14], [15]. Moreover, RGD peptides added to pea epicotyls reduced the production of the phytoalexin pisatin [16], and in shear-stressed *Taxus* cells these peptides negatively affected the alkalization response, as well as the accumulation of both, phenolics and reactive oxygen species (ROS) [17]. Accordingly, cowpea and pea cells treated with RGD peptides display a disturbed CW-PM continuum and decreased expression of cell wall-associated defense responses upon fungal penetration, whereas these effects were not seen when RGE peptides were added. Hence, the RGD-mediated reduction of defense response stimulated fungal penetration and intracellular hyphal growth [3].

IPI-O, an RXLR-dEER effector of the oomycete pathogen *Phytophthora infestans*, disrupts CW-PM adhesions in Arabidopsis through its RGD motif [18]. When the RGD motif in IPI-O is mutated to RGE or RGA the adhesions remain intact [18]. Plant plasma membranes possess high affinity RGD-binding sites, and binding was shown to be saturable, reversible and RGD-specific [15]. IPI-O interacts with these sites in a similar manner, and hence competes with other proteins possessing the RGD motif. A phage display, set up to find proteins interacting with the RGD motif of IPI-O, resulted in two peptides that act as RGD-binding antagonists and lead to the identification of an Arabidopsis legume-like lectin receptor kinase named LecRK-I.9 that binds IPI-O via its RGD motif [19]. Since LecRK-I.9 functions in CW-PM adhesions it is conceivable that LecRK-I.9 is involved in protein-protein interactions with RGD-containing proteins as potential ligands and plays a structural and signaling role at the plant cell surface. As yet, little is known about the function of LecRK-I.9 and other legume-like lectin receptor kinases [20] nor about their natural ligands.

Besides the RGD motif, IPI-O contains another characteristic motif called RXLR, that partially overlaps with RGD (i.e., RSLRGD). This motif is shared by numerous secreted oomycete effector proteins several of which are known to function as race-specific avirulence factor [21]. For two other RXLR effectors – i.e., *P. infestans* Avr3a and *P. sojae* Avr1b – it has demonstrated that the RXLR motif is crucial for transfer of the effector to the host cell [22]–[24]. The effector function of IPI-O is further supported by expression data; *ipiO* expression is not detectable in *in vitro* grown mycelium but induced in germ tubes invading host tissue. During infection of susceptible potato lines expression is highest in the periphery of the lesion where the hyphae ramify in the apoplast and in the sub-peripheral zone where plant cells are being invaded by haustoria, but is lacking in the necrotic center of the lesion and in the sporulating zone [25]. Recently we have shown that *ipiO* is the avirulence (*Avr*) gene *Avr-blb1* that acts in a gene-for-gene manner with *Rpi-blb1*, a late blight resistance (*R*) gene from *Solanum bulbocastanum* which encodes a cytoplasmic NBS-LRR protein [26]. In potato plants carrying *Rpi-blb1*, colonization by *P. infestans* isolates that contain class I variants of IPI-O is blocked [27].

The observation of binding between a *Phytophthora* effector and an Arabidopsis legume-like lectin receptor kinase via the RGD cell attachment motif [19] raised questions about the role of LecRK-I.9 in the interaction with *Phytophthora*. Since, Arabidopsis is a non-host plant for *P. infestans* [28], we made use of another *Phytophthora* species, *P. brassicae*. For the interaction between *P. brassicae* and Arabidopsis various distinct incompatible and compatible isolate-accession combinations have been described, and this pathosystem can be regarded as an attractive model for the concurrent analysis of both host and pathogen [29]–[31]. In this study, we first analyzed expression of *LecRK-I.9* under biotic and abiotic stress and determined the sub-cellular localization of LecRK-I.9. Subsequently, we analyzed the response of Arabidopsis *LecRK-I.9* T-DNA insertion mutants (*lecrk-I.9*) to infection with *P. brassicae* and investigated the role of IPI-O in the infection process by making use of transgenic Arabidopsis lines expressing *ipiO1*. Interestingly, the *lecrk-I.9* mutants and the *ipiO1-*expressing lines showed strikingly similar phenotypes. In both cases, we not only observed gain of susceptibility but also comparable patterns of pathogen- and MAMP-triggered callose deposition. Overall, our observations strongly suggest that LecRK-I.9 plays a role in disease resistance and point toward involvement of the RXLR effector IPI-O in infection processes.

## Results

### *LecRK-I.9* expression is induced in incompatible interactions

Analysis of endogenous *LecRK-I.9* expression in Arabidopsis Col-0 by RT-PCR revealed *LecRK-I.9* transcripts in all tested tissues, i.e., in flowers, siliques, stems, rosette leaves and roots (data not shown). To analyze *LecRK-I.9* expression in more detail we used a transgenic Arabidopsis Col-0 line carrying the reporter construct P*_LecRK-I.9_*-GUS consisting of the promoter region (−1486 to +9 bp) of *LecRK-I.9* fused to the coding sequence of the β-glucuronidase (GUS) gene. Analysis of this line, selected because of a single copy insertion and a representative GUS staining pattern [32], revealed that *LecRK-I.9* is differentially expressed during organ differentiation with very low or no expression in fully differentiated tissues.

**Figure 1:**
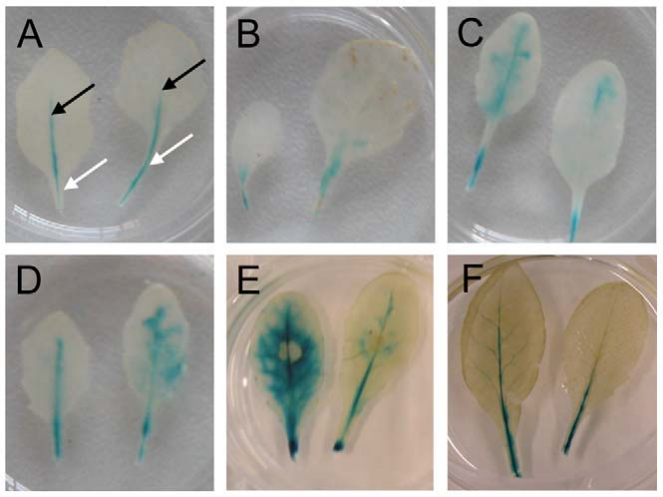
*LecRK-I.9* expression is induced in an incompatible but not in a compatible interaction with *P. brassicae*. GUS activity in leaves of a transgenic Arabidopsis line carrying promoter-GUS construct P*_LecRK-I.9_*-GUS. (A) In mature non-inoculated leaves GUS activity is localized in the petiole (white arrows) and in veins (black arrows). GUS activity in leaves, 6 days post-inoculation (dpi) with the virulent *P. brassicae* isolate CBS686.95 (B), the avirulent *P. brassicae* isolate HH (C), *P. infestans* IPO-0 (D), 4 dpi with *B. cinerea* IMI169558 (E), and 2 days after wounding (F).

We inoculated the P*_LecRK-I.9_*-GUS reporter line with two *P. brassicae* isolates; one that is compatible with Col-0 (isolate CBS686.95) and another one (isolate HH) that is incompatible with Col-0 but capable to infect other Arabidopsis accessions [29]. GUS staining upon inoculation with isolate CBS686.95 was comparable to the staining observed in un-inoculated or mock-treated leaves (Fig. 1A and 1B). In both cases we observed a blue color in the petiole, and in the primary veins of rosette leaves but not in leaf epidermal and mesophyll cells. In contrast, inoculation with HH resulted in a significant increase in GUS activity as early as 1 day post-inoculation (dpi). Fig. 1C shows the GUS activity at 6 dpi. A similar increase, also appearing within 1 dpi, was observed in the non-host interaction of Col-0 with *P. infestans* and upon inoculation with isolate IMI169558 of the necrotrophic grey mold fungus *Botrytis cinerea*, which can infect and colonize Col-0 (shown in Fig. 1D and 1E, respectively, and at 6 dpi). The prominent difference between an incompatible and compatible interaction was also observed by others (C. Balagué and D. Roby, personal communication). They found increased GUS activity within 6 hours after inoculation with the avirulent *Pseudomonas syringae* strain DC3000(*avrRpm1*), while in a compatible interaction with *P. syringae* DC3000 there was only a slight increase in GUS activity. The expression patterns that we observed in the P*_LecRK-I.9_*-GUS reporter line are consistent with expression data retrieved from publicly available repositories [20]. Taken together, these results reveal induction of expression of *LecRK-I.9* upon infection with *B. cinerea* and in incompatible interactions with biotrophic *Phytophthora* and *Pseudomonas* pathogens.

### LecRK-I.9 is localized at the plasma membrane

**Figure 2:**
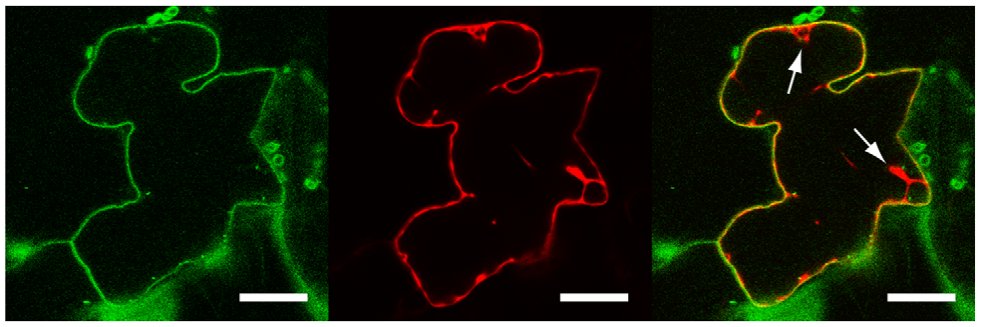
LecRK-I.9 is localized at the plasma membrane. Confocal images of a *N. benthamiana* epidermal cell transiently expressing LecRK-I.9-GFP and mCherry. Single-channel (i.e., GFP left, mCherry middle) and merged fluorescence images (right). Arrows point towards cytoplasmic strands. Scale bars represent 20 µm.

LecRK-I.9 contains a putative transmembrane domain [20], and hence hypothesized to be associated with a membrane. To determine the subcellular localization of LecRK-I.9 we constructed the binary vector pS-LecRK-I.9-GFP that is suitable for transient *in planta* expression of C-terminal GFP-tagged *LecRK-I.9*. Leaves of *Nicotiana benthamiana* were co-infiltrated with *Agrobacterium* strains carrying pS-LecRK-I.9-GFP and pBIN61-mCherry (free mCherry; [33]) and after 2 to 3 days examined by confocal laser scanning microscopy. GFP fluorescence was detected at the cell surface, whereas the red fluorescent signal of free mCherry was found to be distributed in the nucleus and the surrounding cytoplasm, and throughout the cytoplasm lining the cell surface (Fig. 2). Merging the GFP and mCherry images revealed distinct fluorescence of both GFP and mCherry at the cell surface with GFP more peripheral than mCherry (Fig. 2). Co-localization of GFP and mCherry was never observed within the nucleus or in the endoplasmic reticulum network that surrounds the nucleus, nor in cytoplasmic strands (data not shown). GFP fluorescence at the plasma membrane and distinct from free mCherry was reproducible in several independent experiments. This demonstrates that in these transient expression assays LecRK-I.9-GFP is targeted to the plasma membrane and points to association of LecRK-I.9 with the plasma membrane.

### Arabidopsis mutants disrupted in *LecRK-I.9* show gain of susceptibility towards *P. brassicae*

**Figure 3:**
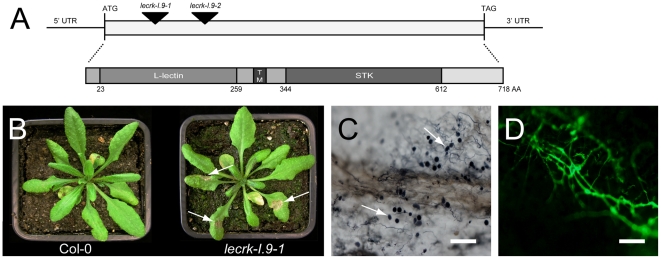
Arabidopsis mutants disrupted in *LecRK-I.9* are impaired in resistance to *P. brassicae*. (A) Schematic representation of *LecRK-I.9* and its gene product. The coding sequence is 2154 nt in length and has one continuous open reading frame. The two mutant lines *lecrk-I.9-1* and *lecrk-I.9-2* have a T-DNA insertion (black arrowheads) at position 176 and 566, respectively, relative to the translational start. The LecRK-I.9 protein contains an extracellular legume-like lectin domain (L-lectin), a transmembrane motif (TM), and an intracellular serine/threonine protein kinase domain (STK). (B) Col-0 and *lecrk-I.9-1* plants 4 days post-inoculation with *P. brassicae* isolate HH. Arrows point at lesions. Colonization of *lecrk-I.9-1* leaves by *P. brassicae* isolate HH visualized by trypan blue staining (C) and *P. brassicae* GFP transformant 155 m revealed by UV epifluorescence (D). Arrows in (C) point at sporangia. Scale bar in (C) represents 100 µm and in (D) 200 µm.

To be able to study the potential function of LecRK-I.9, we obtained two mutant lines with T-DNA insertions just downstream of the translation start site, i.e., *lecrk-I.9-1* and *lecrk-I.9-2* (Fig. 3A). RT-PCR using gene-specific primers confirmed that both *lecrk-I.9* mutant lines lack *LecRK-I.9* expression [32]. Growth behavior and morphology of the two homozygous *lecrk-I.9* mutants and the recipient line Col-0 were comparable (data not shown). Also generation time, seed setting and seed germination were not affected in LecRK-I.9 deficient plants. We then compared the response of the *lecrk-I.9* mutants and Col-0 to inoculation with *P. brassicae*. Isolate HH is incompatible with Col-0 and unable to colonize Col-0 plants. In contrast, plug-inoculation of *lecrk-I.9* plants with isolate HH resulted in lesions that first appeared 2 days after inoculation and expanded further until complete leaf collapse after 7 days (Fig. 3B). Similar results were obtained when Col-0 and *lecrk-I.9* plants were inoculated with zoospores of isolate HH (data not shown). This gain of disease susceptibility was observed in several assays on both *lecrk-I.9-1* and *lecrk-I.9-2*. It is a stable phenotype and the infection efficiency (IE) of isolate HH on the two *lecrk-I.9* lines is on average over 80 percent. Trypan blue staining of *lecrk-I.9* leaves infected with HH revealed heavy tissue colonization and sporulation (Fig. 3C). This was further confirmed by UV fluorescence microscopy in *lecrk-I.9* leaves infected with a GFP-tagged transformant of isolate HH (Fig. 3D) [34]. Comparable disease symptoms and IE were found when *lecrk-I.9* plants were inoculated with *P. brassicae* isolate II, which is – like isolate HH – incompatible with Col-0 (Fig. S1).

To determine the specificity of this gain of disease susceptibility, we performed infection assays with other pathogens. Despite the fact that expression of *LecRK-I.9* is induced in response to infection with *B. cinerea* (Fig. 1E), *lecrk-I.9* did not show stronger disease symptoms compared to Col-0. Moreover, inoculation of *lecrk-I.9* with the non-host pathogens *P. infestans* and *Alternaria brassicicola*, a necrotrophic fungus, did not result in lesion formation and no changes in disease susceptibility were found with respect to the hemibiotrophic fungus *Colletotrichum destructivum*, which forms a compatible interaction with Col-0.

### Overexpression of *LecRK-I.9* leads to developmental effects and enhanced resistance to *P. brassicae*

**Figure 4:**
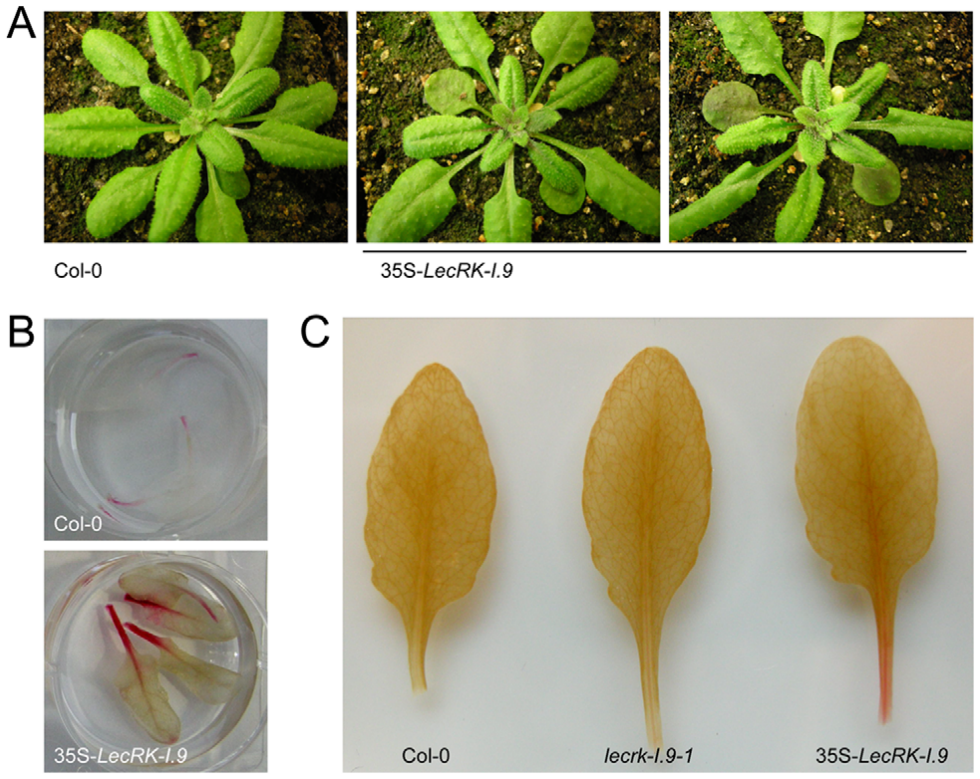
*LecRK-I.9* overexpression in Arabidopsis leads to changes in morphology and accumulation of anthocyanin and lignin. In comparison to the recipient line Col-0, 35S-*LecRK-I.9* lines have more compact rosettes with smaller and slightly wrinkled leaves (A). Anthocyanin (B) and lignin (C) staining in rosette leaves of Col-0 and 35S-*LecRK-I.9* lines.

To further investigate the role of *LecRK-I.9* in the defense response, transgenic Arabidopsis plants were generated that constitutively express *LecRK-I.9*. A construct containing the full-length coding sequence of *LecRK-I.9* under the control of the constitutive cauliflower mosaic virus 35S (CaMV 35S) promoter was transferred to Arabidopsis accession Col-0 via flower-dip transformation. Multiple independent lines were obtained, two of which were selected for further analysis (i.e., C-0123 and C-0126). In comparison to the recipient line Col-0, both 35S-*LecRK-I.9* lines had more compact rosettes with smaller and slightly wrinkled leaves (Fig. 4A), and were shorter in height (data not shown). Moreover, the transgenic lines displayed a substantially higher accumulation of anthocyanin and lignin, a phenomenon not observed in Col-0 or the *lecrk-I.9* lines (Fig. 4B, and C). Anthocyanin pigmentation and lignin deposition was most intense in mature cotyledons, and along the petioles and midribs of young rosette leaves (Fig. 4B, and C).

**Figure 5:**
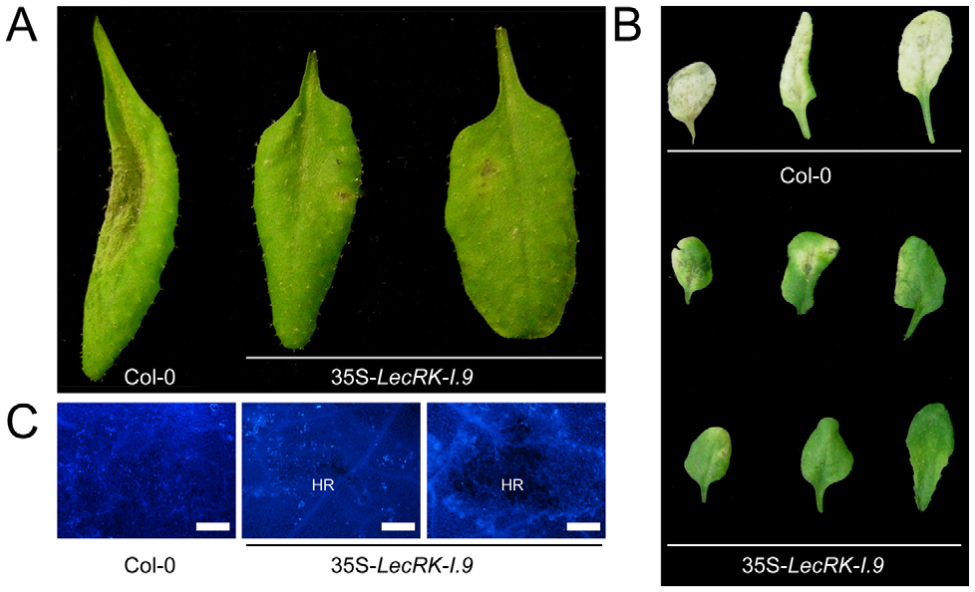
*LecRK-I.9* overexpression in Arabidopsis results in enhanced resistance to *P. brassicae*. Arabidopsis leaves inoculated with plugs (A) or zoospores (B and C) of *P. brassicae* isolate CBS686.95. Pictures were taken 4 days post-inoculation (dpi) (A), 8 dpi (B), and 5 dpi (C). Lesion expansion was observed on Col-0 leaves, but not on leaves from two independent 35S-*LecRK-I.9* lines. In (C) leaves were stained for callose (light blue fluorescence). HR; hypersensitive response. Scale bars in (C) represent 50 µm.

When we challenged the two 35S-*LecRK-I.9* lines and the recipient line Col-0 with *P. brassicae* isolate HH we observed no differences. Col-0 is incompatible with HH and this incompatibility with HH is maintained in the 35S-*LecRK-I.9* lines (data not shown). Col-0 is compatible though with *P. brassicae* isolate CBS686.95 and upon inoculation with this isolate, both with mycelium plugs and by drop inoculation with a zoospore suspension, Col-0 leaves became completely colonized. In contrast, such colonization was not observed in the transgenic 35S-*LecRK-I.9* lines. These plants showed an elevated level of resistance towards this *P. brassicae* isolate (Fig. 5A, and B); there were no or only minor disease symptoms but instead there was a hypersensitive response (HR), including an increase in callose deposition, which was not found in Col-0 (Fig. 5C). The experiment was repeated several times with comparable results. No differences in disease progression were observed between Col-0 and the 35S-*LecRK-I.9* lines upon inoculation with *Botrytis cinerea* (data not shown).

### *lecrk-I.9* and 35S-*ipiO1* lines are gain of susceptibility phenocopies

**Figure 6:**
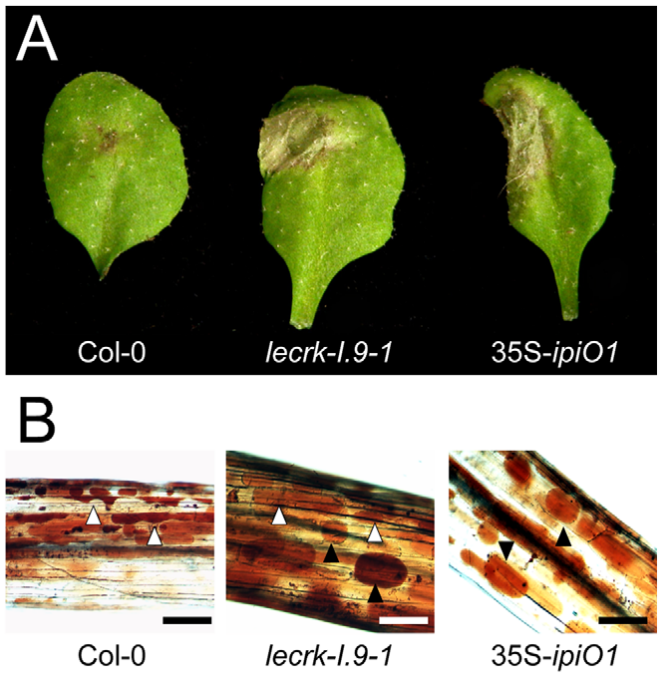
Arabidopsis *lecrk-I.9* and 35S-*ipiO1* lines are phenocopies. Both lines show (A) gain of susceptibility to *P. brassicae,* and (B) defects in cell wall integrity. (A) Leaves inoculated with *P. brassicae* isolate HH 3 days post-inoculation (dpi). (B) Elicitation of plasmolysis in etiolated hypocotyls by treatment with 0.4 M CaCl_2_. Shown are concave (white arrowheads) and convex (black arrowheads) forms of plasmolysis. In Col-0 only a few cells show convex plasmolysis, in *lecrk-I.9* convex shapes occur more frequently whereas in 35S-*ipiO1* the majority of cells is convex. Etiolated hypocotyls were stained with neutral red. Scale bars represent 100 µm.

To examine whether IPI-O functions as an effector that manipulates host defense responses, we generated Arabidopsis transformants carrying a transgene composed of the coding sequence of the *P. infestans ipiO1* gene fused to the constitutive CaMV 35S promoter in a Col-0 background. Transgene expression was confirmed by RT-PCR (data not shown). The transformants developed normal and, with respect to morphology and size, they could not be distinguished from Col-0 plants. Multiple independent transformants (35S-*ipiO1*) were tested for their response to *P. brassicae*. In contrast to Col-0, which, as expected, exhibited full resistance to *P. brassicae* isolate HH, the 35S-*ipiO1* plants displayed clear disease symptoms 3 days after inoculation with HH (Fig. 6A). The foliar lesions developed gradually over time, and leaves were completely wilted after 7 days. This phenotype was observed in several independent experiments, and the IE on the 35S*-ipiO1* plants was comparable (75%) to the IE on *lecrk-I.9* lines. Trypan blue staining of infected leaf material revealed massively intercellular hyphal growth and sporulation similar to what was observed in the infected *lecrk-I.9* lines (data not shown). Comparable disease symptoms and IE were found when the 35S-*ipiO1* plants were inoculated with *P. brassicae* isolate II (Fig. S1). Also with respect to infection with the fungi *A*. *brassicicola*, *C*. *destructivum* and *B*. *cinerea*, and the bacterium *P. syringae* the 35S*-ipiO1* plants responded similar as the two *lecrk-I.9* lines (Table 1). These results show that in response to pathogens the *lecrk-I.9* and 35S-*ipiO1* lines behave as phenocopies.

**Table 1:** Gain of susceptibility to *Phytophthora brassicae* in Arabidopsis Col-0, *lecrk-I.9* and 35S-*ipiO1* lines.

Pathogen	Observed disease phenotype a		
	Col-0	*lecrk-I.9*	35S-*ipiO1*
*Botrytis cinerea* IMI169558	S	S	S
*Colletotrichum destructivum* IMI349061	S	S	S
*Alternaria brassicicola* MUCL20297	R	R	R
*Phytophthora infestans* IPO-0	R	R	R
*Phytophthora brassicae* CBS686.95	S	S	S
*Phytophthora brassicae* HH	R	S	S
*Phytophthora brassicae* II	R	S	S
*Pseudomonas syringae* DC3000(*avrRpm1*)	R	S b	S
*Pseudomonas syringae* DC3000	S	S^+^ b	S^+^

a based on at least three independent experiments; R, resistant; S, susceptible. S^+^, more susceptible.

b C. Balagué and D. Roby, personal communication.

### *lecrk-I.9* and 35S-*ipiO1* lines show changes in the CW-PM continuum

Previously, it was shown that exposing Arabidopsis cells to IPI-O protein, obtained by heterologous *ipiO* expression in *E. coli*, results in disruption of the CW-PM continuum [18]. To investigate if ectopic expression of *ipiO* in Arabidopsis causes the same effect we examined the CW-PM continuum in 35S-*ipiO1* plants. Plasmolysis was induced by soaking the hypocotyls in 0.4 M CaCl_2_. Upon staining with neutral red we observed that in Col-0 the PM readily separated from the CW, but at several points the adhesions between CW and PM were maintained, resulting in pockets that are concave with respect to cells. In contrast, convex forms of plasmolysis were observed in 35S-*ipiO1* plants; the PM quickly separated from the CW reaching a near-spherical shape (Fig. 6C), similar to what was previously observed upon adding IPI-O1 protein to Arabidopsis cell suspension cultures [18]. To exclude that constitutive expression of *ipiO* leads to secondary effects, indirectly causing disruption of CW-PM continuum, we generated stable transformants in which *ipiO1* expression is under control of the alcohol-inducible *alcA* promoter. Similar to the 35S-*ipiO1* transformants, the *alcA-ipiO1* transformants were morphological comparable to the parental line Col-0. After exposure of the *alcA-ipiO1* plants to 0.01% (v/v) ethanol for 30 min, plasmolyzed hypocotyls displayed a phenotype identical that of 35S*-ipiO1* plants: i.e., convex forms of plasmolysis (Table 2; Fig. S2). Hence, we can conclude that IPI-O causes the loss of CW-PM adhesions.

**Table 2:** The RGD motif in IPI-O is required to disrupt CW-PM adhesions *in planta*.

Transgene a	Observed type of plasmolysis b	
	concave	convex
None	x (88%, n = 24) c	
35S-*ipiO1*		x (70%, n = 23) c
35S-*ipiO1* ^RGE^	x	
*alcA*-*ipiO1*		x
*alcA*-*ipiO1* ^RGE^	x	
*alcA*-*ipiO1* ^RGA^	x	

a in Col-0 background;

b based on three independent experiments;

c percentage observed in one experiment out of three with similar outcome; n =  number of examined hypocotyls.

A logic next step was to examine the CW-PM adhesions in the phenocopy *lecrk-I.9* lines. As shown in Fig. 6C, the convex forms of plasmolysis that were observed in 35S*-ipiO1* and *alcA-ipiO1* plants were also found in both knock-out lines, *lecrk-I.9-1* and *lecrk-I.9-2*. It should be noted, however, that the relative number of hypocotyls with cells that showed a convex shape of plasmolysis was much less than in the 35S*-ipiO1* lines. These observations imply that overexpression of *LecRK-I.9* would lead to an increase in the strength of the CW-PM adhesions. Unfortunately, it was not possible to measure this. When we compared plasmolysed cells in hypocotyls from wild-type Col-0 and 35S-*LecRK-I.9* lines no differences were observed and even at higher CaCl_2_ concentration (0.8 M) the shape in the plasmolysed Col-0 cells remained concave.

To determine whether CW-PM interactions are disturbed plant cells challenged with *Phytophthora*, we examined the CW-PM integrity in plasmolysed epidermal cells of *N. benthamiana* – which can be considered as a host plant [35] – shortly after infection with *P. infestans* (i.e., 5-7 hpi). To better visualize penetration, the leaves were inoculated with zoospores of a GFP-labeled *P. infestans* isolate (88069-GFP). Upon infection, we observed a strong detachment between CW and PM in epidermal cells penetrated by *P. infestans*. In contrast, this detachment was not observed in uninfected neighboring cells (Fig. S3).

### *lecrk-I.9* and 35S-*ipiO1* lines are impaired in callose deposition

**Figure 7:**
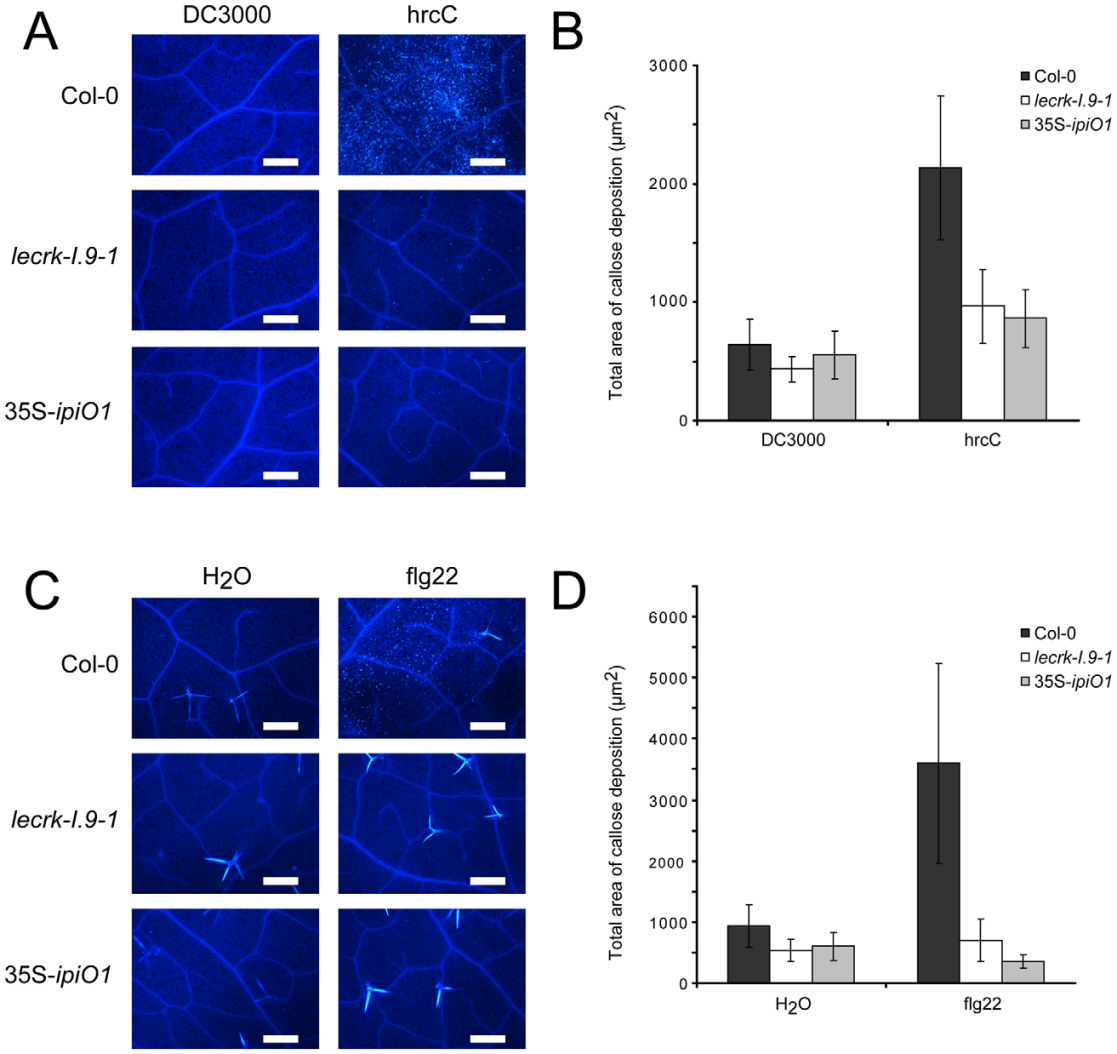
Pathogen- and MAMP-triggered callose deposition is reduced in *lecrk-I.9* and 35S-*ipiO1* lines. (A) Arabidopsis leaves stained with aniline blue after infiltration with *Pseudomonas syringae* DC3000 and hrcC. (B) Average totals of callose deposition (µm2) and associated 95% confidence intervals (CIs) after infiltration with DC3000 and hrcC (n =  >16) (C). Callose deposition after infiltration with flg22. (D) Average totals of callose deposition after treatment with flg22 (µm2) +95% CIs (n =  >10). Scale bars in (A) and (C) represent 50 µm.

The gain of susceptibility and impairment of cell wall integrity suggests that cell wall associated defense response could be affected in the *lecrk-I.9* and 35S-*ipiO1* lines. To investigate this, we analyzed the level of callose deposition in the phenocopy lines after infiltration with *P. syringae*. Strain DC3000 produces effectors that suppress defense in Col-0 and, as a result, callose deposition is decreased [36]. The *P. syringae* hrcC mutant can no longer suppress defense resulting in callose accumulation. Microscopic analysis revealed that DC3000-infiltrated leaves did not (or hardly) display callose deposition in Col-0, and neither in *lecrk-I.9* nor in 35S-*ipiO1* lines (Fig. 7A, and B). As expected, Col-0 leaves infiltrated with the *P. syringae* hrcC mutant displayed a significant increase of callose deposition but in contrast, this increase was not observed in hrcC infiltrated leaves of *lecrk-I.9* and 35S-*ipiO1* plants (Fig. 7A, and B). Similarly, infiltration with the oligopeptide flg22 resulted in extensive callose deposition in Col-0, but hardly any callose deposition was found in *lecrk-I.9* and 35S-*ipiO1* lines (Fig. 7C, and D). This points at a defect in MAMP-triggered callose deposition in both, the *lecrk-I.9* and 35S-*ipiO1* lines.

### The RGD motif in IPI-O is a determinant of the phenotypic changes

**Figure 8:**
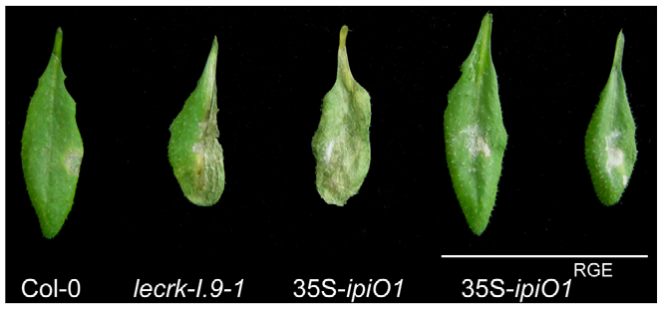
The RGD motif in IPI-O is a determinant of the phenotypic changes. Arabidopsis transgenic lines expressing *ipiO1* with a RGD-to-RGE targeted mutation show no gain of susceptibility to *P. brassicae* HH. Arabidopsis leaves inoculated with *P. brassicae* strain HH 5 days post-inoculation.

We then addressed the role of the RGD motif in IPI-O and questioned if RGD is a determinant of the phenotypic changes observed in Arabidopsis upon ectopic *ipiO1* expression. We therefore generated transgenic lines carrying *ipiO1* constructs with site-directed mutations changing RGD to RGE or RGA. Two independent 35S-*ipiO1* ^RGE^ lines, one *alcA*-*ipiO1* ^RGE^ line and two *alcA*-*ipiO1* ^RGA^ lines were tested in a plasmolysis assay. In none of these lines a convex type of plasmolysis was observed, and as in Col-0 the CW-PM adhesions remained intact (Table 2). This is in agreement with the previous results that showed that in cell suspension cultures exogenously added mutant forms of IPI-O1 (IPI-O1^RGE^ and IPI-O1^RGA^) had no disrupting effect [18]. Subsequently, we examined how a RGD-to-RGE mutation in IPI-O1 affects susceptibility to *P. brassicae*. As described above Col-0 exhibited full resistance to *P. brassicae* isolate HH whereas the leaves of 35S-*ipiO1* plants were completely wilted leaves 5 days post-inoculation. On the two independent 35S-*ipiO1* ^RGE^ lines no lesions appeared and the phenotype was comparable to the incompatible interaction between Col-0 and HH (Fig. 8). Taken together, these results show that the RGD cell adhesion motif in IPI-O is crucial for disruption of the CW-PM continuum, as well as the gain of susceptibility to *P. brassicae*.

## Discussion

Membrane-spanning receptor proteins are supposed to play important roles in sensing alterations at the plant cell wall and, subsequently, to mediate response reactions [37]. Legume-like lectin receptor kinases (LecRKs) are regarded as candidates for monitoring cell wall integrity, and are likely functional in responses to various stresses. Up-till now only few reports have addressed the role of LecRKs in plant-pathogen interactions. Arabidopsis has a family of 45 LecRKs divided over nine clades and a few singletons [20]. This study revealed that one of these LecRKs, i.e., LecRK-I.9, is a novel membrane-associated *Phytophthora* resistance component. In incompatible interactions LecRK-I.9 plays a crucial role in arresting growth of the pathogen. In compatible interactions the pathogen exploits an effector carrying a RGD cell-attachment motif to disturb the CW-PM continuum, possibly by targeting the anchor protein LecRK-I.9.

LecRK-I.9 was initially identified in a phage display as a protein interacting with the *P. infestans* RXLR-dEER effector IPI-O via the RGD motif present in IPI-O [19]. In this study we first analyzed *LecRK-I.9* gene expression and observed that under normal growth conditions *LecRK-I.9* is expressed throughout the plant at a low level with higher levels of expression during organ differentiation. In compatible interactions with biotrophic pathogens *LecRK-I.9* expression did not change. Infected and mock-treated leaves showed the same low basal level of *LecRK-I.9* expression. In contrast, *LecRK-I.9* expression was much higher when biotrophic pathogens encountered a strong hypersensitive response (HR) and were thus unable to colonize the leaves. *LecRK-I.9* expression was also strongly increased in a non-host interaction with *P*. *infestans,* which shows an HR reminiscent of an incompatible interaction, and in lesions resulting from infection with the necrotrophic pathogen *B. cinerea*, in which cells are committed to programmed cell death [38]. Taken together, these expression patterns suggest that LecRK-I.9 functions in plant defense.

Strong evidence for a role for LecRK-I.9 in defense was obtained by making use of *LecRK-I.9* T-DNA insertion mutants (*lecrk-I.9*) and *LecRK-I.9* overexpressing lines (35S-*LecRK-I.9*). Their response to infection with various pathogens demonstrated that LecRK-I.9 is crucial for resistance of Arabidopsis to *P. brassicae*. Previously, it was reported that resistance in Arabidopsis to *P. brassicae* is not dependent on salicylic acid (SA), jasmonic acid (JA) and ethylene (ET) defense signaling pathways; i.e., disease resistance was maintained in mutants deficient in these pathways [29]. In contrast, mutants deficient in PAD2 lost resistance and the *PAD2* gene is thus crucial for full-resistance to *P. brassicae* [29]. *PAD2* encodes a γ-glutamylcysteine synthetase (GSH1), which is the first enzyme in the glutathione biosynthesis pathway [39]. Lower glutathione (GSH) levels in the *pad2-1* mutant were found to be correlated with reduced accumulation of glucosinolates; toxic plant compounds which could have an negative effect on *P. brassicae* [40], [41]. So far, a link between GSH and lectin receptor kinases has not been reported. Intriguingly, in human cells a glutathione redox potential appears to regulate integrin-mediated cell adhesion [42] and hence, it is worth to investigate whether such a glutathione redox potential also influences cell adhesions and CW-PM adhesions in plants.

Apart from the change in disease phenotype both the insertion mutants and *LecRK-I.9* overexpressing lines, showed certain developmental effects. In the 35S-*LecRK-I.9* lines this was visible with the naked eye. In comparison to the parental Col-0 line, 35S-*LecRK-I.9* plants were smaller in size, had wrinkled leaves and displayed accumulation of anthocyanins and lignin suggesting that these lines are some-how stressed. As yet, we have no clue if there is a correlation between the increase in anthocyanin and lignin and the enhanced resistance towards *P. brassicae*. Both anthocyanin and lignin are known to be induced after elicitation of basal defense. An increase in anthocyanin was shown to inhibit susceptibility of potato tubers to *Pectobacterium carotovora* ssp. *carotovora* [43], whereas Arabidopsis plants with an impaired monolignon synthesis exhibit enhanced susceptibility towards various bacterial and fungal pathogens [44]. The 35S-*LecRK-I.9* plants also showed an increase in callose deposition upon inoculation with *P. brassicae*, whereas this increase was not found in Col-0. This suggests that LecRK-I.9 directly or indirectly triggers the enhancement of callose deposition, which is regarded as an early cellular marker of response upon cell wall damage, and recognition of MAMPs and pathogen elicitors.

The developmental effect that we observed in the *lecrk-I.9* plants was more subtle. The morphology of the plants was not affected but at the cellular level we did find a phenotype that is in line with what we expected based on previous studies. Gouget et al. (2006) [19] who selected LecRK-I.9 in a phage display aimed at identifying proteins interacting with the RGD cell attachment motif in the *P. infestans* effector IPI-O, showed that the seven amino acid peptides resulting from the phage display disrupted the CW-PM adhesions in Arabidopsis hypocotyls as visualized by convex forms of plasmolysis. Since these peptides correspond to sequences in the extracellular domain of LecRK-I.9 we assumed that this disruption was due to competition with the natural ligands of LecRK-I.9 thereby disabling the function of endogenous LecRK-I.9. Also in *lecrk-I.9* lines the function of LecRK-I.9 is disabled and indeed, the CW-PM adhesions seem to be slightly reduced. These observations support the hypothesis of Gouget et al. (2006) [19] that LecRK-I.9 functions in protein-protein interactions to mediate adhesions between the CW and PM.

LecRK-I.9 was identified as a protein potentially interacting with IPI-O [19]. The fact that, similar to LecRK-I.9 peptides, endogenously added IPI-O has the capacity to disrupt CW-PM adhesions [18] urged us to investigate the effect of ectopic expression of *ipiO1* on CW-PM adhesions. As shown in Fig. 5C *in planta* expression of *ipiO* results in disturbance of the CW-PM continuum in an RGD dependent manner thereby demonstrating that the RGD cell attachment motif has to be intact to cause the disturbance. In the 35S-*ipiO1* lines the convex form of plasmolysis was much stronger and more frequently observed than in the *lecrk-I.9* lines. Expression analysis in *lecrk-I.9* showed that in some tissues other clade I *LecRK* genes have increased expression levels compared to Col-0 suggesting that the lack of LecRK-I.9 is compensated by other LecRKs. This redundancy likely explains the difference between 35S-*ipiO1* lines and *lecrk-I.9* in the plasmolysis assays. Most interestingly, the 35S-*ipiO1* lines were also found to display enhanced disease susceptibility to *P. brassicae* and thus behaved as phenocopies of *lecrk-I.9*. This phenocopy behaviour was also observed with respect to callose deposition: neither *lecrk-I.9* nor 35S-*ipiO1* lines accumulate callose upon pathogen- and MAMP treatment. Similarly, infiltration with *P*. *syringae* hrcC, resulted in callose deposition in Col-0 but not in the *lecrk-I.9* and 35S-*ipiO1* lines, and this is in agreement with the increase in severeness of disease symptoms caused by *P*. *syringae* in the phenocopy lines.

The effector gene *ipiO1* was isolated from *P. infestans*, a pathogen that infects potato and tomato, but can not infect Arabidopsis. IPI-O belongs to the enormous reservoir of RXLR-dEER effectors present in all *Phytophthora* species analyzed so far [45]. The diversity among RXLR-dEER effectors is extremely high; not only between species but also within a species. For *P. infestans* and *P. sojae* many races are described that show differential interactions with cultivars of potato and soybean, respectively. We now know that the various resistance proteins present in those cultivars recognize different RXLR-dEER effectors from the pathogen [21]. Based on our current knowledge on the diversity in RXLR-dEER effectors it seems unlikely that *P. brassicae*, the species that infects Arabidopsis, contains an *ipiO* homologue. Nevertheless, *P. brassicae* may have a RGD-containing effector that targets LecRK-I.9 in Arabidopsis, but this should be revealed by genome sequencing of *P. brassicae*.

Evidence that pathogen effectors function by manipulating the host is accumulating. Suppression of defense is an effective mechanism that often requires entry of effectors into host cells. The RXLR-dEER motif that is shared by numerous oomycete effectors, including IPI-O, functions as a host cell targeting motif [22], [23], [24]. Indeed, for a few RXLR effectors it has been shown that the RXLR motif mediates transport into the plant cell and that they function inside the cell as suppressors of defense reactions. So far there is no experimental proof that IPI-O suppresses defense in its natural occurrence, i.e. the *P. infestans*-potato interaction. However, based on its activity as AVR factor [27] we have strong indirect evidence that, similar to other RXLR effectors, IPI-O ends up inside the host cell. The matching R protein is Rpi-blb1, an intracellular NBS-LRR protein that upon recognition of most IPI-O variants confers resistance to late blight in potato. This AVR activity of IPI-O, together with its activity unraveled in the current study, points to, at least, a dual function of IPI-O in the host-pathogen interaction. One function is executed when IPI-O resides extracellular and requires the RGD motif, whereas the other function operates intracellularly and is confined to the C-terminal domain of the effector protein. Host cell targeting, most likely mediated by the RXLR motif, can be considered as a third function.

**Figure 9:**
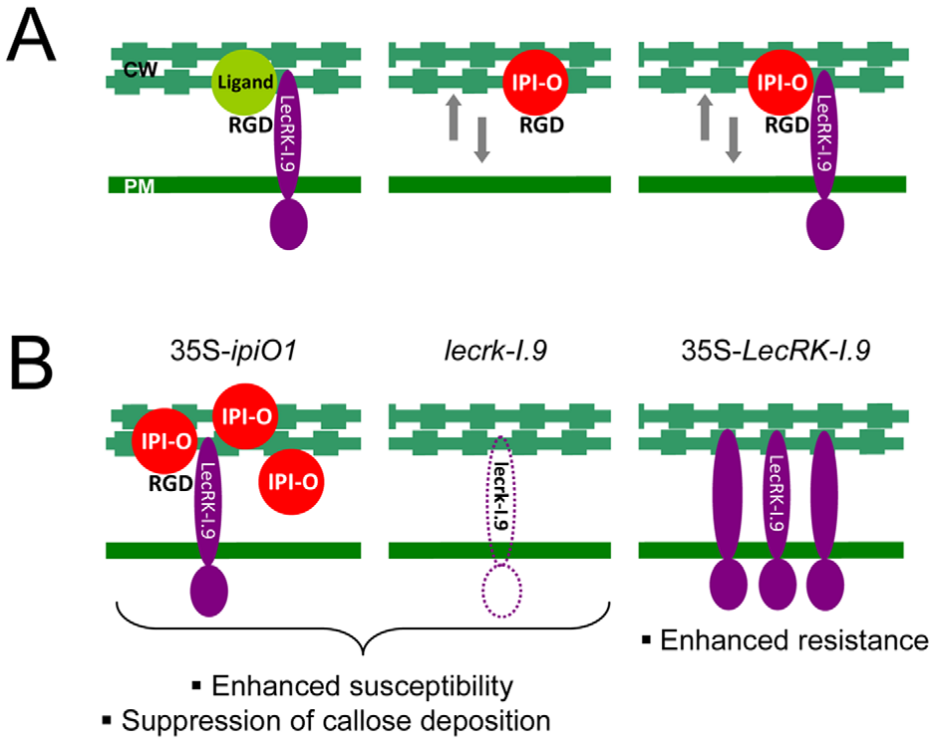
*P. infestans* effector IPI-O and its putative effector target LecRK-I.9. (A) Models depicting the membrane-associated LecRK-I.9 and the influence of IPI-O activity on adhesions between cell wall (CW) and plasma membrane (PM). LecRK-I.9 supposedly interacts with RGD-containing extracellular ligands (left panel). Addition of the RGD-containing effector IPI-O alters the CW-PM continuum (middle), and presumably binding of IPI-O to LecRK-I.9 further disrupts this continuum (right). The putative activity of IPI-O inside the plant cell is not included in these models. (B) Observed changes in the Arabidopsis phenocopy lines 35S-*ipiO1* and *lecrk-I.9* (left and middle panel, respectively), and the *LecRK-I.9* overexpressing lines (right panel).

In our model shown in Fig. 9A, we hypothesize that LecRK-I.9 is an RGD-binding protein that interacts with extracellular ligands. This interaction then results in activation or repression of kinase activity – mediated by the intracellular domain of LecRK-I.9 – leading to regulation of normal plant development. We further hypothesize that *Phytophthora* uses LecRK-I.9 as a gateway to establish infection and that one of the functions of the RGD-containing protein IPI-O is to mediate this early step in the infection process. The other anticipated functions of IPI-O are more downstream in the process and are not included in the model. The model is reminiscent of animal host-pathogen interactions. Also some animal pathogens make use of receptors present in the plasma membrane to gain entry and there are several examples where integrin receptors that bind RGD-containing proteins produced by the pathogen play a role in this process. Obviously, the role of these integrins is not to help pathogens to enter the host cell, but to regulate normal developmental processes that require ‘inside-out’ and ‘outside-in’ signaling over the plasma membrane and we anticipate that the same is true for LecRK-I.9. Based on our results we propose that in plants challenged by *Phytophthora* pathogens loosening of CW-PM adhesions is a ubiquitous step in the infection process. This is supported by the finding that these adhesions are disrupted in *N. benthamiana* leaf cells invaded by germ tubes of *P. infestans* but not in neighboring cells (Fig. S3). As shown previously [18], [19] and in the current study, IPI-O by itself can loosen the CW-PM adhesions and the presumed interaction of IPI-O with LecRK-I.9 via the RGD motif may further disrupt these adhesions. This model explains why *lecrk-I.9* and 35S-*ipiO1* lines show gain of susceptibility and behave as phenocopies, and also explains why overexpression of *LecRK-I.9* enhances resistance (Fig. 9B).

## Materials and Methods

### Plant material and growth conditions

Arabidopsis plants were grown in soil or *in vitro* on solid MS medium (4.4 g/l Murashige and Skoog salts (Duchefa), 0.5% (w/v) sucrose and 1% (w/v) plant agar). Arabidopsis was grown in a conditioned growth chamber at 21–22°C with a 16 h photoperiod and at a relative humidity of 75–80%. Transgenic plants and T-DNA insertion lines were in Col-0 background. The Arabidopsis T-DNA insertion mutants *lecrk-I.9-1* (SALK_042209), and *lecrk-I.9-2* (SALK_024581) were obtained from the European Arabidopsis stock centre NASC (http://arabidopsis.info), and analyzed as described by Gouget (2005) [32]. *Nicotiana benthamiana* plants were grown under standard greenhouse conditions.

### Pathogen growth and infection assays

*Botrytis cinerea* strain IMI169558 and *Alternaria brassicicola* strain MUCL20297 were grown and maintained at 22°C on malt agar and potato dextrose agar plates, respectively. Infection assays of Arabidopsis with *B. cinerea* and *A. brassicola* were performed as described by Van Esse et al. (2007) [46]. *Colletotrichum destructivum* strain IMI349061 was propagated at 22°C on Mathur's agar plates and inoculum was prepared as previously described [47]. Arabidopsis leaves were drop-inoculated with a conidial suspension (1*10^6^ spores ml^−1^), and subsequently incubated at 22°C in trays covered with lids to maintain high humidity. *Phytophthora infestans* strain IPO-0, and the GFP-transformant 88069-GFP were grown on rye agar medium supplemented with 20 g l^−1^ sucrose at 18°C [48] and zoospores were isolated as described in Champouret et al., 2008 [27]. *P. brassicae* strains HH, II, CBS686.95 and the HH GFP-transformant 155 m [33] were grown at 18°C on 10% V8-juice agar plates [49], and zoospores were obtained as described in Bouwmeester and Govers, 2009 [31]. Inoculation was performed by placing plugs of young mycelium (Ø 5 mm) or 10 µl drops of a zoospore suspension (1*10^5^ zoospores ml^−1^) on the abaxial leaf surface. Inoculated plants were kept in trays covered with lids to maintain a high humidity and placed in the dark, and placed in a growth chamber with a 16 h photoperiod at 18°C and a RH of 75%. The first day the trays were kept in the dark. After two days the mycelial plugs were removed from plants to stop the facilitation of nutrition from the agar medium. *Pseudomonas syringae* pv. *tomato* strains were grown on King B agar supplemented with the appropriate antibiotics at 28°C. Arabidopsis leaves were spray-inoculated with bacterial suspensions of 1*10^9^ colony-forming units (cfu) ml^−1^, and incubated as earlier described [50]. Infection efficiencies (IEs) were calculated as percentages of successful infections relative to the total number of inoculations (n>72). All infection assays were performed at least 3 times.

### Plasmid construction and plant transformation

For cloning *ipiO1* (GenBank: L23939.1) in binary vectors *ipiO1* fragments were PCR amplified from the plasmids pPIN18-c, pMBP-IPIO1D56A and pMBP-IPIO1D56E, respectively, using primers IPIOXHO-F and MAL-R (Table S1) [18]. PCR fragments were purified and digested with *Xho*I and *Pst*I to release a 550 bp fragment containing the coding region of *ipiO1*, starting immediately after the signal peptide cleavage site, and ligated into the *Xho*I/*Pst*I digested vector pRH80 [51]. Fragments containing the *ipiO1* coding region fused to the TPI-II terminator sequence were released by digesting with *Xho*I and *Eco*RI, purified and ligated into *Xho*I/*Eco*RI-digested binary vector pRH90 [51] thereby fusing the *ipiO1* coding sequence to the tobacco *PR1a* signal peptide sequence. The resulting binary plasmids were named pRW100, pRW101 and pRW102 with the latter two carrying RGD-tripeptide mutations E56A and D56E, respectively. Amplicons obtained by PCR amplification on pRW100, pRW101 and pRW102 with the primers PstPR1a and PR1 were digested by *Pst*I and ligated into the *Pst*I-digested vector pACN (Table S1) [52]. These plasmids were digested with *Hin*dIII, and the fragments were ligated into the *Hin*dIII*-*digested vector binSRNACatN [52]. In the resulting binary plasmids pRW110, pRW111 and pRW112, the *ipiO1* coding sequence is fused to the ethanol-inducible promoter of the *alcA* gene.

For cloning the full-length coding sequence of *LecRK-I.9* (GenBank: NM_125423.2) driven by the 35S CaMV promoter in a binary vector, a PCR was performed on BAC clone F15L12.17 with primers pK60300-s and pK60300-as or pK60300ms-as (Table S1). PCR fragments were cloned in pENTR/D-TOPO. LR recombination enabled cloning into binary vector pK2GW7 (http://www.psb.ugent.be/gateway) [53] and pSOL2095 resulting in plasmids pK-35S-LecRK-I.9 and pS-35S-LecRK-I.9-GFP, respectively. Cloning steps were verified by sequencing. Binary vectors were transformed to *Agrobacterium tumefaciens* strain GV3101 or AGL1 and cultured on medium containing the appropriate antibiotics. *Arabidopsis thaliana* accession Col-0 was transformed by the floral dip method [54]. Transformed plants were selected on MS agar with 50 mg/l kanamycin. Rooted seedlings were subsequently transferred to potting soil. Multiple independent lines were selected. Agroinfiltration assays were performed as previously described [27]. Leaves of 4–5 week old *N. benthamiana* plants were co-infiltrated in a 1∶1 ratio with *A. tumefaciens* suspensions of OD 0.5–1.0, and were analyzed by confocal laser scanning microscopy, 2–3 days after infiltration.

### Staining techniques and microscopy

For GUS histochemical staining, Arabidopsis tissues were immersed and vacuum-infiltrated in X-gluc staining buffer [50 mM phosphate buffer pH 7.0, 0.1% (v/v) Triton X-100, 1 mM 5-bromo-4-chloro-3-indolyl-β-D-glucorunide (X-gluc, Biosynth, Staad, Switzerland), 1 mM K_3_Fe(CN)_6_*3H_2_O), 1 mM K_4_Fe(CN)_6_*3H_2_O and 10 mM EDTA] and incubated overnight at 37°C. Chlorophyll was removed by incubation in a 50–96% ethanol series. GUS stained samples were examined at low magnification under brightfield illumination. Pictures of GUS-stained tissues shown in this paper are representative results from at least three independent experiments. Neutral red staining and plasmolysis of etiolated Arabidopsis seedlings was performed as previously described with minor modifications [19]. Etiolated seedlings were incubated in a 0.05% neutral red solution for 30 min and rinsed afterwards in distilled water. Plasmolysis was induced by the addition of 0.4 M CaCl_2_. Pictures were taken after 5 min of plasmolysis. Trypan blue staining was performed as described earlier [55]. Lignification was visualized as earlier described by Mohr and Cahill (2007) [56]. In brief, leaves were cleared in ethanol and incubated overnight in a phloroglucinol-ethanol mixture, and subsequently placed for 5 min in 20% HCl and washed with water. To visualize anthocyanin pigmentation, leaves were incubated overnight in the dark at 4°C in 80% methanol containing 1% HCl. Brightfield and fluorescence microscopy was performed with a Nikon 90i epifluorescence microscope (Nikon, Amstelveen, The Netherlands). GFP and mCherry were visualized using a GFP-B (EX 460–500, DM 505, BA 510–560) and a TRITC (EX 540/25, DM 565, BA 606/55) filter cube. Confocol laser scanning microscopy was performed using a Zeiss Confocor 2–LSM 510 (Carl Zeiss, Jena, Germany). GFP and mCherry were excited with an Argon (488 nm line) and HeNe (543 nm line) laser, respectively. Fluorescence was captured through the band-pass emission filters BP505-530 (GFP) and 600–650 nm (mCherry). To visualize callose deposition, Arabidopsis leaves were harvested approximately 18 h after inoculation with bacteria or infiltration with 10 µM flg22, cleared overnight in an ethanol series (70–96%) and stained with 1% (w/v) aniline blue in 150 mM K_2_HPO_4_ (pH 9.5) for 1 h. Stained leaves were mounted in 50% glycerol, and fluorescent callose deposits were viewed using epifluorescence microscopy (DAPI filter; EX340-380, DM 400, BA 435-4850). Images of randomly selected fields were captured using a Nikon DS-5Mc digital camera and processed with ImageJ software (http://rsb.info.nih.gov/ij) to calculate the total area (µm^2^) of callose deposits. Values in Fig. 7B and D are the average of at least 16 and 10 microscopic fields, respectively. Error bars represent 95% confidence intervals (CIs).

### RNA isolation and RT-PCR

Total RNA of Arabidopsis was isolated and purified using a NucleoSpin RNA Plant kit (Macherey**-**Nagel). RT-PCR was performed on a ABI7300 Real Time PCR system (Applied Biosystems) with use of a SYBR Green I qPCR kit (Eurogentec) and gene-specific primers (Table S1).

### Accession numbers

IPI-O1; Q01918

LecRK-I.9; Q9LSR8/Q56XH0

## Supporting Information

Figure S1 Arabidopsis *lecrk-I.9* and 35S-*ipiO1* lines show gain of susceptibility to *P. brassicae* II. Leaves inoculated with *P. brassicae* isolate HH and II 3 days post-inoculation.
(0.09 MB PDF)

Figure S2 Arabidopsis hypocotyls show concave plasmolysis in Col-0, but convex forms in 35S-*ipiO1* and *alcA-ipiO1* lines. (A,B) Schematic representation of the constitutive and alcohol-inducible *ipiO1* gene expression constructs, respectively. (C) Etiolated Arabidopsis hypocotyls after plasmolysis using 0.4 M CaCl_2_. Scale bars represents 50 µm.
(4.38 MB PDF)

Figure S3 Penetration of *N. benthamiana* epidermal cells by *P. infestans* reduces cell wall-plasma membrane adhesions. Image of epidermal cells after plasmolysis with 0.4 M CaCl_2_. Arrows indicate positions where the plasma membrane (PM) has pulled away from the cell wall (CW). Note that the cell penetrated by a germ tube (*) shows a strong detachment between CW and PM. In the uninfected neighboring cells Hechtian strands (h) and CW-PM connections (c) are maintained. gc  =  germinating cyst, v =  vacuole. Scale bar represents 10 µm.
(0.14 MB PDF)
