# Supplementary figures and images for: The Lectin Receptor Kinase LecRK-I.9 Is a Novel Phytophthora Resistance Component and a Potential Host Target for a RXLR Effector
Source: PLoS Pathog. 2011 Mar 31;7(3):e1001327. doi: 10.1371/journal.ppat.1001327 (PMC3068997; doi:10.1371/journal.ppat.1001327)

HH

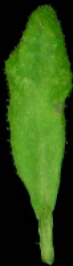

Col-0

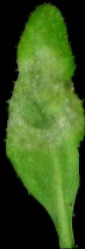

*lecrk-1.9-1*

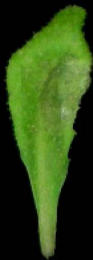

35S-*ipiO1*

II

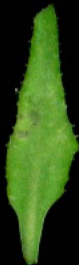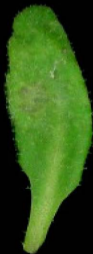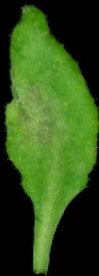

Supplement: Figure S1 — Arabidopsis lecrk-I.9 and 35S-ipiO1 lines show gain of susceptibility to P. brassicae II. Leaves inoculated with P. brassicae isolate HH and II 3 days post-inoculation. (0.09 MB PDF) [file ppat.1001327.s001.pdf]

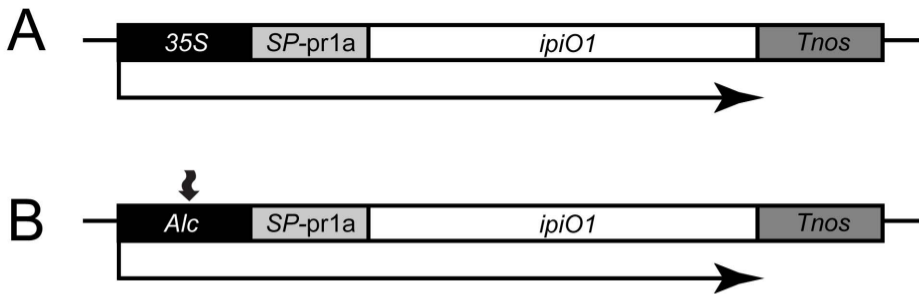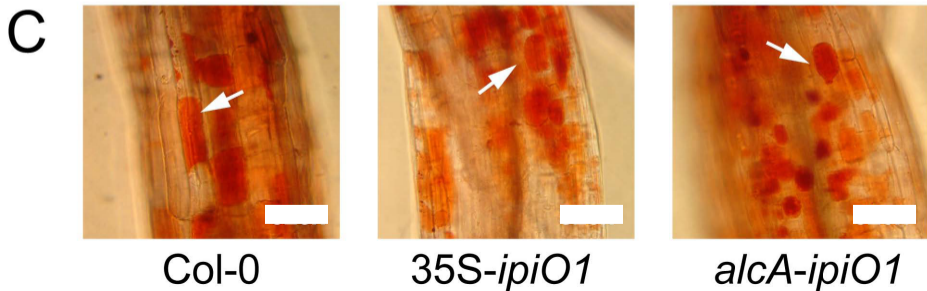

Supplement: Figure S2 — Arabidopsis hypocotyls show concave plasmolysis in Col-0, but convex forms in 35S-ipiO1 and alcA-ipiO1 lines. (A,B) Schematic representation of the constitutive and alcohol-inducible ipiO1 gene expression constructs, respectively. (C) Etiolated Arabidopsis hypocotyls after plasmolysis using 0.4 M CaCl2. Scale bars represents 50 µm. (4.38 MB PDF) [file ppat.1001327.s002.pdf]

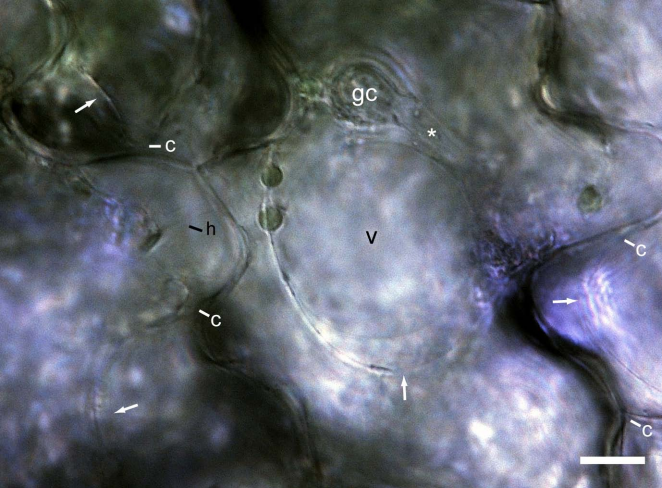

Supplement: Figure S3 — Penetration of N. benthamiana epidermal cells by P. infestans reduces cell wall-plasma membrane adhesions. Image of epidermal cells after plasmolysis with 0.4 M CaCl2. Arrows indicate positions where the plasma membrane (PM) has pulled away from the cell wall (CW). Note that the cell penetrated by a germ tube (*) shows a strong detachment between CW and PM. In the uninfected neighboring cells Hechtian strands (h) and CW-PM connections (c) are maintained. gc = germinating cyst, v = vacuole. Scale bar represents 10 µm. (0.14 MB PDF) [file ppat.1001327.s003.pdf]
